# Supplementary material for: Climate change threatens Chinook salmon throughout their life cycle
Source: Commun Biol. 2021 Feb 18;4:222. doi: 10.1038/s42003-021-01734-w (PMC7892847; doi:10.1038/s42003-021-01734-w)
Supplement: Supplementary file 2 — Supplementary Information [file 42003_2021_1734_MOESM2_ESM.pdf]

Supplementary Information for
Chinook salmon populations face challenges from climate change across their life cycle

Crozier, Lisa C., Burke, Brian J., Chasco, Brandon E., Widener, Daniel L., and Zabel, Richard W.

Contents

Supplementary Tables 2
Supplementary Table 1. Data sources for climate projections. 2
Supplementary Table 2. Biological data sources. 3
Supplementary Table 3. Environmental data for model fitting 4
Supplementary Table 4. Distributions used as priors. 5
Supplementary Table 5. Freshwater model comparison. 6
Supplementary Table 6. Marine survival model selection table.Supplementary.... 7

Supplementary Figures 8
Supplementary Fig. 1. Prior and posterior distributions of parameters. 8
Supplementary Fig. 2. Covariance of environmental variables. 9

Supplementary Methods 10
Submodels for individual life stages 10
Spawner-to-parr and parr-to-smolt stages 10
Downstream survival ($S_{mainstem}$) 12
Smolt-to-adult return ocean survival (S_{SAR}) 13
Upstream survival ($S_{upstream}$) 15
Aggregation of downstream and upstream survival models to annual time step for climate scenarioscenarios 16
Downstream survival ($S_{mainstem}$) 16
Upstream survival ($S_{upstream}$) 17

References 20

Supplementary Tables

Supplementary Table 1. Data sources for climate projections. Source information for climate trends for each environmental variable in the life-cycle model. RCP 4.5 and 8.5 scenarios were produced for the IPCC 5th Assessment Report (AR5), while the A1B and B2 scenarios were produced for the IPCC 4th Assessment Report (AR4).

Variable, Source	Emissions scenario	GCMs/ scenario	Time series/ scenario	Comments
Air temperature and snowpack[1]	RCP 4.5, 8.5	10	10	We aggregated daily max and min into seasonal mean and April 1 snowpack from the grid cell that includes the upper Middle Fork Salmon River
Flow, Salmon, ID[1]	RCP 4.5, 8.5	10	40	We aggregated daily data to seasonal flows using the Multivariate Adaptive Constructed Analogs (MACA) downscaling method, followed by 4 hydrological models
Flow LGR[1]	RCP 4.5, 8.5	10	40	We aggregated daily data to seasonal flows using the Multivariate Adaptive Constructed Analogs (MACA) downscaling method, followed by 4 hydrological models
Stream temperature LGR[2]	RCP 4.5, 8.5	10	40	We used air temperature, snowpack and flow to model stream temperature in the Lower Granite Pool for input into the COMPASS model
Flow BON[3,4]	A1B, B2	2	2	Although only 2 fully downscaled time series were available to run the daily submodel, we used the high correlation between BON and LGR to select representative years from the upstream survival simulations to follow the trends in LGR time series (see Appendix S3)
Stream temperature BON[3,4]	A1B, B2	2	2	
Sea surface temperature[5]	RCP 4.5, 8.5	26	26	We utilized monthly output for 2x2 degree grid cells, selecting the area included in the arc polygon (Johnstone and Mantua 2014) or the 4 degree grid cell off WA coast (Lat 46-48, Lon -124--126)
Upwelling[6]	RCP 8.5	1	80	We utilized monthly output from 40 members of the Community Earth System Model Large Ensemble Project (CESM-LE). Each ensemble member begins from a slightly different initial atmospheric temperature in 1920, is subject to historical radiative forcing through 2005, and RCP 8.5 radiative forcing from 2006 to 2100.

Supplementary Table 2. Biological data sources. Data sources for each life stage survival estimate, years included in analysis, sample sizes (N) for stage survivals or uncertainty estimates that we analyzed for this study. Data used in fitting the equations shown in the text is available in the data archive. See references for additional information. Note that years listed for SAR span outmigration and adult return years. Outmigration years were 2000-2015.

Life stage	Years	N
Spawner abundance[7,8]	1998-2016	33542
Stributary (s2)[9]	2000-2014	171004
Smainstem (s2)[10]	2000-2014	~1,600,000
SAR (s3, s0)[11]	2000-2017	122,415
Supstream[12]	2004-2017	7553

Supplementary Table 3. Environmental data for model fitting. Abbreviations; USGS-U.S. Geological Survey, PRISM-Parameter-elevation Regressions on Independent Slopes Model, USACE, U.S. Army Corps of Engineers. *ICOADS: International Comprehensive Ocean-Atmosphere Data Set https://icoads.noaa.gov/

Life stage	Covariate	Abbreviation	Source	Years
Stributary	Seasonal mean flow at Salmon, ID Gage 13302500	F_{SAL}	USGS[13]	1960-2018
Stributary	Seasonal mean air temperature, Middle Fork Salmon River, ID: 4-km grid cell 44.4068N, 115.3520W	T_{SAL}	PRISM[14]	1960-2018
Smainstem	Mean temperature and flow in Lower Granite pool (April-June), proposed action in EIS (DOE/EIS-0529)	T_{LGR}, F_{LGR}	USACE[15]	1929-2008
Supstream	Mean temperature and flow, Bonneville Dam scroll case (April-June)	T_{BON}, F_{BON}	USACE[16]	1980-2018
	Mean temperature, Bonneville Dam water quality monitoring station		USACE[16]	1980-2017
Ssar	Extended Reconstructed SST dataset (ERSST), polygon defined by Johnstone and Mantua 2014	SSTarc	*ICOADS[17]	1900-2017
	ERSST: 4 × 4-degree region off Washington coast	SSTwa	*ICOADS[17]	1900-2017
	Bakun upwelling index for 45N, 125W	U	NOAA[18]	1930-2017

Supplementary Table 4. Distributions used as priors for the hypermeans of the hierarchical model. The standard deviation was expressed as the precision for the actual model, where the precision=1/variance.

	Mean of the hypermean			SD of the hypermean		
Parameter	Distribution	Mean/ Minimum	Standard deviation/ Maximum	Distribution	Minimum	Maximum
p1	Normal	6	5	Uniform	0.001	10
p2	Uniform	0	10	Uniform	0.2	3
c1	Normal	0	10	Uniform	0.2	3
c2	Normal	0.3	10	Uniform	0.3	10
βT	Uniform	-10	10	Uniform	0.002	10
βF	Uniform	-10	10	Uniform	0.002	10

Supplementary Table 5. Freshwater model comparison. Models that included covariates (*e*) that affected productivity ($ln(R_t/S_t) = p + c \times lnS_t + e_t$) are labeled p_2, while covariates that affected capacity ($ln(R_t/S_t) = p + (c + e_t) \times lnS_t$) are labeled c_2. T refers to air temperature and F refers to flow in the Salmon River Basin, described in Supplementary Table 3 (T_{SAL} and F_{SAL}). The season selected for each variable is shown for each model. We compared models based on the differences in expected log pointwise predictive density (ΔELPD) and their standard errors (se). The top models are shown here. The two selected freshwater covariate models (p2Tsummer+Ffall and p2Fsummer) were combined with two selected SAR models (Supplementary Table 6) for a total of three LCM models, which are labeled with superscripts in this table (Model 1, Model 2, and Model 3 in the main text).

Model	ΔELPD	se
p2Ffall	0	0
c2Fsummer	-0.1	0.3
p2Tsummer+Ffall[1,3]	-0.2	0.1
p2Fsummer[2]	-0.2	0.2
c2Tsummer+Fsummer	-0.2	0.3
c2Ffall	-0.2	0.3
c2Tsummer+Ffall	-0.3	0.2
p2Tsummer+Fsummer	-0.3	0.3

Supplementary Table 6. Marine survival model selection table. SAR models selected (in bold) for climate projections in relation to variable importance. Additional columns show the specific model that includes the designated variable with the lowest AIC (Top model 1) and the next lowest AIC (Top model 2). The two selected SAR models were combined with two selected freshwater covariate models (Table S4) for a total of three LCM models (Model 1, Model 2, and Model 3) labeled with superscripts in this table. The season for each index was January-March (WIN), April-June (SPR), or July-September (SUM). Environmental covariates: sea surface temperature (SST), coastal upwelling index (U), North Pacific Gyre Oscillation (NPGO), the multivariate ENSO index (MEI), Oceanic Niño Index (ONI), and the North Pacific Index (NPI).

In-river fish

Rank	Variable	Importance	Topmodel 1	Topmodel 2
1	SSTwa.sum	0.24	**SSTarc.win + SSTwa.sum** [1,2]	SSTwa.sum + ONI.spr
2	SSTarc.spr	0.24	SSTarc.spr + SSTwa.aut	**SSTarc.spr + U.spr** [3]
3	SSTarc.win	0.18	SSTarc.win + SSTwa.sum	SSTarc.win + NPI.spr
4	SSTwa.spr	0.16	SSTwa.spr + NPI.sum	SSTwa.spr + MEI.sum
5	NPI.spr	0.13	NPI.spr + ONI.sum	SSTarc.win + NPI.spr
6	U.spr	0.09	SSTarc.spr + U.spr	SSTarc.win + U.spr

Transported fish

Rank	Variable	Importance	Topmodel 1	Topmodel 2
1	SSTarc.win	0.14	**SSTarc.win** [1,2]	SSTarc.win + SSTwa.sum
2	SSTwa.sum	0.12	**SSTwa.sum** [3]	SSTarc.win + SSTwa.sum
3	SSTwa.win	0.1	SSTwa.win + NPI.sum	SSTwa.win + NPGO.aut
4	SSTwa.spr	0.1	SSTwa.spr + U.win	SSTwa.spr + NPGO.sum
5	NPI.win	0.09	MEI.sum + NPI.win	SSTwa.sum + NPI.win
6	NPGO.aut	0.09	SSTwa.win + NPGO.aut	NPGO.aut

Supplementary Figures

Supplementary Fig. 1. Prior and posterior distributions of parameters.

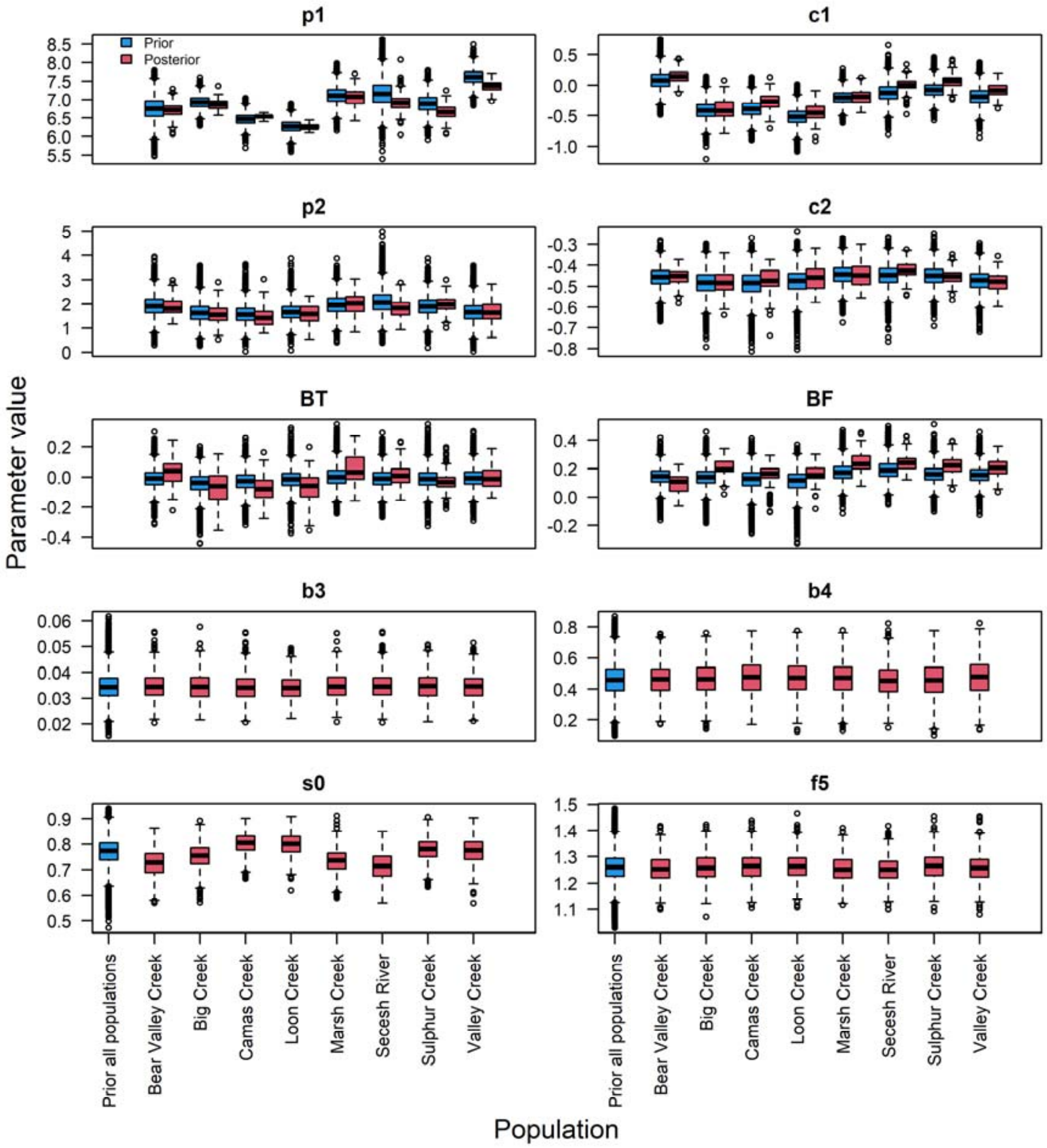

The prior (blue) and posterior (red) distributions of parameters resulting from the calibration process. Parameter names are from equation (S2) and Appendix S2. The populations were most differentiated in the spawner to parr parameters (p1 and c1) and ocean survival (s0). The other maturation parameters were similar to Zabel, et al.[19]. Boxes show the interquartile range across simulations, while the whiskers extend to 1.5 times the interquartile range. The horizontal line shows the median value.

Supplementary Fig. 2. Covariance of environmental variables.

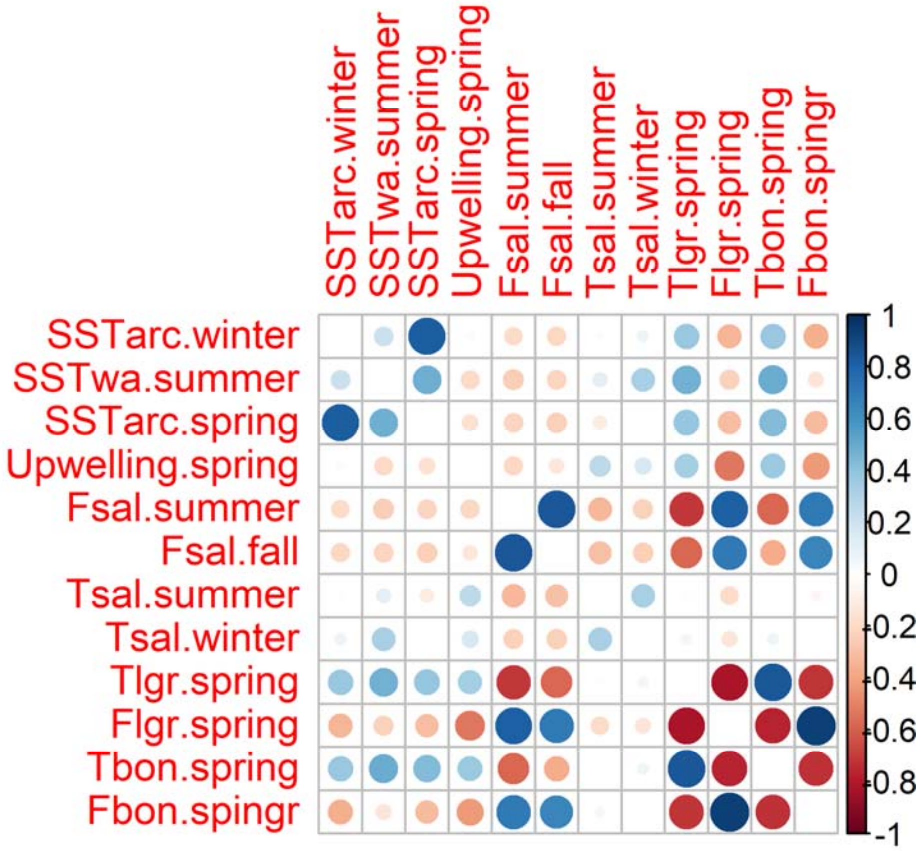

Descriptions of covariates and acronyms are shown in Supplementary Table 2. Correlation coefficients were generated by TMB model builder based on data available from 1966-2015.

Supplementary Methods

Submodels for individual life stages

We used a modified 2-stage Gompertz equation to model recruits per spawner in a manner that accounted for density dependence. The Gompertz equation is:

$$ln\left(\frac{Sp_{k,t+x}}{Sp_{k,t}}\right) = p + c \cdot ln\left(Sp_{k,t}\right) + \varepsilon_t \quad [S1]$$

where spawners from year t and population k are $Sp_{k,t}$, recruits are the returning adult progeny in year $t + x$ ($x = 3,4,5$), p is a productivity term and c is a capacity term, and ε_t is a normally distributed error term. This can be converted to a 2-stage model simply by multiplying the individual Gompertz equations together, $G = G_1 \times G_2$, where the subscripts 1 and 2 reflect life stages. We further modified the equation by allowing p and c from the individual life stages to be functions of environmental factors (flow and temperature).

Spawner-to-parr and parr-to-smolt stages

We fit adult recruits per spawner for eight populations in a hierarchical Bayesian framework using multiple likelihood equations which reflected stages that could be directly compared with data (Table S1). We fit a 2-stage Gompertz function (G_1 and G_2) for spawner-to-smolt productivity combined with survival rates estimated independently for later stages. The recruitment function was:

$$Ln(Sp_{k,t+x} / Sp_{k,t}) \sim G_{1,k}(Sp_{k,\ ,t},\ p_1, c_1) \times G_{2,k}(N_{1,k,t}, p_2, c_2, T_{t+1}, F_{t+1}) \times$$

$$\beta eta(S_{mainstem,t+2}, \sigma_{t+2}) \times \beta eta(S_{sar,t+2},\ \tau_{t+2}) \times \beta eta(S_{upstream,t+x,k}\ \upsilon_{t+x}) \times S_{prespawn} \quad [S2]$$

where N_1 is the number of fish alive at the end of year 1, T and F are seasonal mean temperature and flow, respectively, in the parr year (t + 1). Descriptions of environmental covariates are in Table S2. Smolt migration through the mainstem Snake and Columbia Rivers ($S_{mainstem}$) and ocean (S_{sar}) survival are tied to juvenile migration year (t + 2). Each βeta distribution has a standard error associated with the original model estimate ($S_{mainstem}$: σ, S_{sar}: τ, $S_{upstream}$: υ). Upstream migration survival ($S_{upstream}$) is associated with the year of spawning migration, $t + x$ (x = 3, 4, 5).

We fit equation S2 using maximum likelihood, assuming ln($Sp_{k,t+x}$ / $Sp_{k,t}$) (recruits/spawner) was normally distributed. In our model fits, parr abundance (N_1) was a latent variable, but $G_{2,k}(N_{1,k,t},T_t,F_t) = ln(S_{tributary,k,t})$, where k represents the specific population. We solved equation (S1) simultaneously with equation (S2), from PIT-tag survival data using

$$G_{2,k}(N_{1,k,t}, \mathrm{T}_t, F_t) = \ln(N_{2,k,t+1}/N_{1,k,t})0$$
$$= p_k + c_k \ln(N_{1,k,t}) + B_{F,k}\mathrm{F}_t + B_{T,k}\mathrm{T}_t + \varepsilon_{k,t} \quad [S3]$$

Each population had its own coefficients for temperature B_T and flow B_F, but they were drawn from their respective hypermeans, as were the rest of the G_1 and G_2 parameters.[20] $\mathbf{B}_k \sim \mathrm{N}(\mathbf{B},\boldsymbol{\sigma})$, where $\mathbf{B}_k$ is the vector of population-specific coefficients $p_{1,k}$, $p_{2,k}$, $c_{1,k}$, $c_{2,k}$, $\beta_{T,k}$, $\beta_{F,k}$, and **B** and **σ** are vectors of hypermeans and their standard deviations.

We conducted these analyses using JAGS software via the R2JAGS package [21] in R [22]. For each candidate model, we generated three separate Markov chain Monte Carlo (MCMC) chains of 5 million iterations each. We discarded the first half of each chain. The remaining samples were thinned to every 500th sample, producing 3 chains with length 5000 samples, for a total 15,000 maximum likelihood estimates for each parameter. We assessed convergence of the chains using the Gelman and Rubin's convergence diagnostic (gelman.diag function in the coda package). The multivariate potential scale reduction factor was <1.0125 for all initial models (6 models, in which covariates included summer temperature and one of spring, summer or fall flow, and covariates were incorporated into either the productivity or the capacity terms). We also examined Heidelberger and Welch's convergence diagnostics. To ensure all chains were long enough, we re-ran all models with a single chain that was 15 million iterations. All of the parameters in all models passed this diagnostic, except for the two models that included both summer temperature and summer flow. They still had one parameter each that failed the Heidelberger and Welch convergence diagnostic (at eps=0.1 and pvalue=0.05). Although visual examination of the chains and density distributions looked satisfactory, we did not use these models in further analysis.

Priors for the hypermeans were either normally (p1, c1, c2) or uniformly (p2, Bt, Bf) distributed with means informed by non-Bayesian maximum likelihood estimates (Table S3). We used a uniform distribution for all standard deviations of the hypermeans. In essence, we selected priors that widely spanned all of the population estimates for each parameter. Initial values were near the middle of the prior hypermean distributions, and 0

for the environmental coefficients. The posterior distributions resulting from these model fits were used as priors in the tuning step described below.

To determine the best environmental covariates for the parr to smolt stage (G_2), we compared the estimated pointwise predictive error of alternative models using a leave-one-out cross-validation method. We applied the Pareto smoothed importance sampling method for Bayesian models implemented using the LOO package in R, loo_compare function, [23] Covariates included summer air temperature (Tsu), summer flow (Fsu), and fall flow (Ffall).

Multiple models had similar fits, i.e., the difference in expected log pointwise predictive density (ELPD), or ΔELPD, was less than the standard error (se) on estimates of ΔELPD (Table S4). However, the covariates they included had different climate trends. We selected models that were most illustrative in potential for divergent responses to climate change. We therefore explored implications for life-cycle projections of two different freshwater covariate models. In Model 1, T_t was summer air temperature and F_t was fall stream flow, while model 2 included summer stream flow only ($B_T = 0$).

Downstream survival ($S_{mainstem}$)

We used the COMPASS model to estimate juvenile migration survival through the Lower Snake and Columbia Rivers from Lower Granite to Bonneville Dam ($S_{mainstem}$), as well as to estimate arrival day at Bonneville.[24] COMPASS models downstream travel time, passage route, and survival of salmonid smolts. The model comprises eight dams and eleven riverine reaches, from Lower Granite pool on the Snake River to Bonneville Dam tailrace on the Columbia River. Each dam and riverine reach has associated algorithmic equations that use environmental covariates including flow, temperature, and spill to predict fish survival and migration rate in riverine reaches and the proportion of fish that use the spillway, turbine or bypass passage route at dams.

Survival at dams is based on the proportion of fish that pass via each passage route, with survival through each route based on estimates of survival from dam passage studies. The model runs on a sub-daily timestep, and uses environmental inputs on a daily level to update its equations for each timestep.

Fish are added at the top segment of the COMPASS model (Lower Granite pool) according to an empirically-based release distribution, and then the model advances sequentially via timesteps, moving the fish downstream using the migration rate equation and applying mortality in each timestep according to mortality rate equations. The version of the COMPASS model used for this analysis was calibrated for Snake River

stocks of Chinook salmon, using PIT-tag data from 1998 through 2017 [10]. Separate survival and migration rate equations were calibrated for riverine reaches of the Snake vs. the Columbia River.

We created a linear model of median arrival timing at Lower Granite Dam versus mean water temperature and flow in Lower Granite Pool. We used estimated historical arrival distributions from 1998-2019 using PIT tag data (methods from[25], data from[11]) to estimate the historical dates of median smolt arrival at Lower Granite Dam. For each year 1998-2019 we created averages of flow and water temperature for each month February through May as well as bimonthly and whole-season averages (data from[16]). We tested all of these average temperature and flow variables as predictors of median arrival timing, and selected the best-fitting model that contained a single flow predictor and a single temperature predictor. The best-fitting model used mean flow in March and mean temperature in April:

$$M = 151.99 - 0.0798 \times F_{MAR} - 3.049 \times T_{APR}$$

where M is the median date of smolt arrival at Lower Granite Dam, F_{MAR} (P=0.017) is mean March flow in Lower Granite Pool, and T_{APR} (P=0.018) is mean April water temperature in Lower Granite Pool. The adjusted R^2 for the model over the 1998-2019 dataset was 0.38.

We created an average across-year distribution of daily smolt arrival timing at Lower Granite Dam using the estimated historical distributions from 1998-2019. We centered the yearly distributions based on their individual median arrival days and then averaged daily arrival proportions across the distributions. For a given hypothetical year in the prospective model runs this average distribution was shifted according to the median arrival day predicted by the median arrival timing model. The resulting average arrival distribution was then used as the daily release distribution at Lower Granite Dam for the modeling of downstream smolt survival and migration.

Smolt-to-adult return ocean survival (S_{SAR})

We used a mixed-effects logistic regression model to determine effect of date of ocean entry and environmental covariates on the probability that an individual fish would return as an adult to Bonneville Dam. Our estimate of survival for fish entering on date j in year t was a combination of fixed effects for average survival (μ) and a vector of coefficients ($\boldsymbol{\beta}$) related to observed annual ocean conditions in year t ($\mathbf{x}_t$), as well as random effects related to year (w_t), day of year (v_j), and the interaction between year and day (h_{jt}).

$$logit(s_{jt}) = \mu + \boldsymbol{\beta}\mathbf{x}_t + v_j + w_t + h_{jt} \qquad \text{[S4]}$$

Date and year random effects were described by an auto-regressive lag 1 (AR1) process, where ρ^v and ρ^w were the correlations between date and year time-steps, respectively, and ϕ^v and ϕ^w were standard deviations of the random effects.

$$v_j \sim Normal(\rho^v v_{j-1}, \phi^v) \quad [S5]$$
$$w_t \sim Normal(\rho^w w_{t-1}, \phi^w) \quad [S6]$$

The interaction between date and year was a two-dimensional AR1 process, where $\mathbf{h}_t$ was a vector of date effects within year *t*, ρ^t was the correlation between vectors in years *t* and *t - 1*, and $\mathbf{\Sigma}$ was the covariance matrix describing AR1 process for date within a year,

$$\mathbf{h}_t \sim \text{MVN}(\rho^t \mathbf{h}_{t-1}, \mathbf{\Sigma}) \quad [S7]$$

The covariance matrix, $\mathbf{\Sigma}$, is described by the variance on the diagonal of the matrix, and the correlation between day effects on the off diagonal,

$$\Sigma(\text{j}, \text{j} + \delta) = \frac{\sigma_h^2}{\left(1-(\rho^j)^2\right)(1-(\rho^t)^2)} \left(\rho^j\right)^\delta \quad [S8]$$

where δ is the number of days between j and $j + \delta$, ρ^j is the correlation between days, and σ_h^2 is the variance in the day by year interaction effect.

We considered all four seasonal indices of seven environmental covariates: SST averaged over the entire migration route (SST_{ARC}), SST within 4°C along the Washington coast (SST_{WA}), and the strength of upwelling (coastal upwelling index, cui). We also examined the North Pacific Gyre Oscillation (NPGO), the multivariate ENSO index (MEI), Oceanic Niño Index (ONI), and the North Pacific Index (NPI). We compared models with 0, 1, and 2 ocean covariates separately for in-river and transported fish. Models with no covariates were not supported, and all of the top models also included a day effect and a day × year interaction.

Fish that migrated volitionally through the hydropower system (in-river fish) were treated separately from fish transported through the system on barges. We selected two high-ranking models for in-river and transported fish that captured the variety of trends available from GCM projections. To select models, we first calculated model weights as the relative likelihood of the model as a proportion of the sum of likelihoods across all models, using Akaike information criterion AIC, [26] We then calculated the importance of individual variables (the sum of weights of all models that include that variable).

The four variables with highest importance based on AIC weights for in-river migrating fish were SST_{ARC} (winter and spring) and SST_{WA} (spring and summer), shown in Supplementary Table 6. Spring NPI and upwelling (U) were next for in-river fish. For transported fish, SST_{ARC} and SST_{WA}, were also the most important variables, followed by NPI and NPGO.

In considering which models to include in our simulations, we wanted to account for the possibility that some indices of ocean productivity could increase over time. We accomplished this by selecting another relatively high-performing model that included coastal upwelling. We thus selected two high-ranking models for in-river and transported fish (modeled separately), both of which captured the variety of trends projected for climate indices. All models included a random day effect and a random day × year interaction.

Upstream survival ($S_{upstream}$)

The adult migration survival model is described more fully by Crozier et al. 2020. Briefly, we used generalized additive mixed models (GAMMs) to evaluate the effects of covariates on spring/summer Chinook salmon survival. We split migration through the hydrosystem into two reaches: a Columbia reach from Bonneville to Ice Harbor Dam, and Snake reach from Ice Harbor to Lower Granite Dam. We fit separate models by reach because of distinct characteristics of temperature and flow in the Columbia and Snake rivers.

The continuous covariates we tested included temperature (T) and flow (F) on the day of entry into each reach, harvest (catch, C) recorded in the Columbia River Zone 6 fishery in the Columbia reach, and cumulative temperature accumulation (degree days i.e., mean temperature times travel time) from Bonneville to Ice Harbor Dam ($T_{BO\text{-}IH}$) as a predictor of survival through the Snake reach. We fit each covariate using thin plate regression splines[27] with a maximum of 4 knots for temperature and flow, and 3 knots for catch and cumulative temperature. We also included four factor variables: population, whether the fish was of hatchery (H) or wild origin, whether the fish migrated downriver as a juvenile in-river or was barged (J), and age of adult return (A). Finally, we included a random effect for migration year (y).

We compared all combinations of covariates and selected the model with the lowest AIC. The final model for survival through the Columbia reach ($S_{columbia}$) that includes smoothers for temperature, s(T), flow, s(F) and catch, s(C) was

$$S_{columbia} \sim s(T_{BO}) + s(F_{BO}) + s(C) + H + J + A + y \qquad [S9]$$

The final model for the Snake reach was

$$S_{snake} \sim s(T_{BO\text{-}IH}) + s(T_{IH}) + s(F_{IH}) + H + A + y. \quad [S10]$$

The final model for the Salmon reach was

$$S_{salmon} \sim s(T_{BO\text{-}LG}) + s(T_{Sal}) + s(F_{Sal}) + H + A + y. \quad [S11]$$

Aggregation of downstream and upstream survival models to annual time step for climate scenarios

Downstream survival ($S_{mainstem}$)

To link the sub-daily timestep used by the COMPASS model to the environmental conditions produced in our climate scenarios and explore a broader combination of temperatures and flows than was observed in the historical record, we replicated and modified the historical time series of conditions in the mainstem Columbia and Snake River to meet certain conditions. We first aggregated each of the 80 years of historical daily time series (1929-2008) used by the Army Corps of Engineers ACOE, [15] into spring mean temperatures and flows (1 April-30 June) at Lower Granite Dam for each year (hereafter, the "annual mean"). This was the 80-year time series entered into the covariance matrix model used to simulate the stationary climate.

We then identified the range of mean spring temperatures and flows generated in the climate scenarios. From the annual means, we selected 8 flows (20, 50, 100, 150, 200, 250, 300, and 350 kcfs) and 10 temperatures (9-18°C) to span this range, and created an 8 × 10 matrix of flow-by-temperature combinations. We then modified each year in the USACE historical daily time series by adding (or subtracting) a fixed number of degrees to all days until the mean temperature in the daily record matched the specified mean in the matrix.

Daily flows were scaled by a fixed proportion until they matched the specified flow in each cell of the matrix. Thus each of the 80 cells in the matrix contained 80 replicate years adjusted to meet the specified mean annual flow and temperature criteria. For each of these 80 × 80 = 6400 time series of daily temperatures and flows, the COMPASS model estimated annual survival and the arrival timing distribution at Bonneville Dam.

The distribution of juvenile arrival dates at Lower Granite Dam used to initiate these COMPASS runs was based on the average proportion of smolts arriving per day at Lower Granite Dam from 1998 to 2019. The median of this distribution varied in simulation runs, but the overall shape was constant. We fit a linear regression model of median arrival day per year 1998-2019 vs. annual metrics of water temperature and flow at Lower Granite Dam.

We compared models that included monthly means from February through May as well as bimonthly and whole-season averages (February-May). We selected the best-fitting model by AIC that contained a single flow predictor and a single temperature predictor ($D_{LGR} = 151.99 - 0.0798 \times F_{MARCH} - 3.049 \times T_{APRIL}$). For each simulation year in the COMPASS climate grid, we used this model to predict the median day of arrival in that year, and shifted the overall arrival distribution such that its median matched the predicted median.

Flow, spill, reservoir elevation, water temperature, and dissolved gas for this study were all modeled by the USACE as the *Preferred Alternative* for the NOAA 2020 Biological Opinion [2]. It is worth noting that the *Preferred Alternative* stipulates turbine replacements at Ice Harbor, McNary, and John Day Dams with substantially lower fish mortality than the existing turbines; this reduction in turbine mortality was modeled in COMPASS.

These runs used a universal transportation start date of 20 April at all three transport dams: Lower Granite, Little Goose, and Lower Monumental. After this date, all fish predicted to enter the bypass system at these dams were considered transported by the COMPASS model. The COMPASS model predicts the proportion of fish passing a dam that will enter the bypass system as a function of percent spill, flow, and potentially also day of year or water temperature (depending on the dam). They were removed from the river at the transport point and added to the tailrace of Bonneville Dam 2 days later. A uniform survival rate of 0.98 during transportation was assumed. In each simulation, COMPASS produced arrival time distributions for in-river and transported smolts at Bonneville Dam, which were then input into the SAR model.

Upstream survival ($S_{upstream}$)

To simulate upstream survival under differing climate scenarios, we also needed to model arrival date at Bonneville Dam and travel time through the hydrosystem. We used a 2-dimensional mixture model to recreate the bimodal distribution of spring and summer-run populations comprising the Snake River spring/summer Chinook ESU:

$$D_{BON} \sim p \times N(\mu_{sp}(E),\sigma_{sp}) + (1- p) \times N(\mu_{su}(E),\sigma_{su}) \quad [S12]$$

We simulated the entire ESU at once for each simulation year, where D_{BON} is the arrival day at Bonneville Dam, p is the proportion of spring-run adults (sp), and 1 - p is the proportion of summer-run adults (su). For each run, the mean μ was modeled as a linear function of a single environmental covariate, E, while the standard deviation σ was a constant. To determine the best covariate for E, we compared monthly and bi-monthly mean flows and temperatures at Bonneville Dam from March to June as predictors of mean arrival day through model selection. The best model by AICc included April-May mean flow for spring run and April mean flow for summer run.

We accounted for uncertainty in parameter estimates by drawing each parameter from a multivariate normal distribution based on the coefficient covariance matrix in each simulation year. Six populations included in the LCM models were treated as spring-run (Bear Valley, Big Creek, Camas Creek, Loon Creek, Marsh Creek, and Sulphur Creek) and two populations were treated as summer-run (Secesh River and Valley Creek), following the analysis of Crozier, et al. [28]

For travel time, we used the mixture model described in Crozier et al.[29] Hourly temperatures and flows at each fishway, tailrace, and reservoir influenced mean travel time through each segment of the migration under each climate scenario. We then applied the conditions associated with each fish throughout its migration to the GAMM to estimate survival under each scenario. For all non-environmental covariates, we randomly sampled with replacement from observed distributions in the 2004-2017 PIT data.

We used these model chains to predict survival under simulated climate scenarios. The climate scenarios consisted of daily time series generated by the Bonneville Power Administration (BPA[30] with temperature modeled using methods described by Yearsley[4]). Historical climate was represented by a 70-year reference period (1929-1998), which was then perturbed using the hydrid-delta method[31] with mean monthly temperatures and flows from GCMs for the 2040s.

Two GCMs had been selected by BPA to span the outcomes of greatest concern for hydrosystem planning: a warm/dry scenario (ECHO_G with emissions scenario B1, "dry") and a hotter/wet scenario (MIROC 3.2 with emissions scenario A1B, "wet"). See Hamlet, et al. [31] for additional information on naturalized routed flows and climate model selection.

We thus had 3 climate scenarios (historic, wet, dry) each consisting of a 70-year

time series of daily mean temperatures and flows throughout the hydrosystem. We looped through each of these scenarios 10 times, with timing and survival models for 100 fish per year to account for heteroscedasticity. Each of these 2100 simulation years thus produced an annual survival probability for spring-run and summer-run fish separately, based on their initial bimodal arrival times at Bonneville Dam.

We tied these annual survival estimates to our life-cycle model by summarizing the environmental conditions of each simulation year with the annual mean April-June temperature and flow conditions at Lower Granite Dam. We treated the 2100 simulation years as independent representations of annual survival. We binned annual temperature and flow conditions in the same manner as for juvenile mainstem survival, and randomly drew one adult survival simulation year from the appropriate bin for each time step in the life-cycle model.

References

1 Chegwidden, O. S. *et al.* How do modeling decisions affect the spread among hydrologic climate change projections? Exploring a large ensemble of simulations across a diversity of hydroclimates. *Earth's Future* **https://doi.org/10.1029/2018EF001047**, 7, doi:10.1029/2018ef001047 (2019).

2 NMFS, National Marine Fisheries Service. Endangered Species Act Section 7(a)(2) Biological Opinion and Magnuson-Stevens Fishery Conservation and Management Act Essential Fish Habitat Consultation. Consultation for the Continued Operation and Maintenance of the Columbia River System. NMFS, Portland, Oregon. Available at https://www.fisheries.noaa.gov/resource/document/biological-opinion-operation-and-maintenance-fourteen-multiple-use-dam-and. (2020).

3 Brekke, L., Kuepper, B. & Vaddey, S. Climate and Hydrology Datasets for Use in the RMJOC Agencies' Longer-Term Planning Studies: Part 1 - Future Climate and Hydrology Datasets. (2010).

4 Yearsley, J. R. A semi-Lagrangian water temperature model for advection-dominated river systems. *Water Resources Research* **45**, W12405, doi:10.1029/2008wr007629 (2009).

5 Alexander, M. A. *et al.* Projected sea surface temperatures over the 21st century: Changes in the mean, variability and extremes for large marine ecosystem regions of Northern Oceans. *Elementa-Science of the Anthropocene* **6**, 9, doi:10.1525/elementa.191 (2018).

6 Brady, R. X., Alexander, M. A., Lovenduski, N. S. & Rykaczewski, R. R. Emergent anthropogenic trends in California Current upwelling. *Geophysical Research Letters* **44**, 5044-5052, doi:10.1002/2017gl072945 (2017).

7 Idaho Department of Fish and Game, Oregon Department of Fish and Wildlife & Washington Department of Fish and Wildlife. *Snake River ESU Spring Summer Chinook Natural Origin Spawner Abundance Dataset (1949-2017)*, 2018).

8 Nez Perce Tribe East Fork South Fork Salmon River summer Chinook and Secesh River summer Chinook, Natural Origin Spawner Abundance Dataset (1957-2017). (Protocol and methods available at ttps://www.cbfish.org/Document.mvc/Viewer/P165414. Personal communication with Mari Williams, NOAAF NWFSC/OAI 2019, 2019).

9 Lamb, J. J. *et al.* Monitoring the migrations of wild Snake River spring/summer Chinook salmon juveniles: survival and timing, 2017. (Report of the National Marine Fisheries Service to the Bonneville Power Administration. Portland, Oregon. , 2018).

10 Faulkner, J. R., Widener, D. L., Smith, S. G., Marsh, T. M. & Zabel, R. W. Survival estimates for the passage of spring migrating juvenile salmonids through Snake and Columbia River dams and reservoirs, 2017. (Draft report of the National Marine Fisheries Service to the Bonneville Power Administration. Portland, Oregon. Available at https://www.nwfsc.noaa.gov/contact/display_staffprofilepubs.cfm?staffid=1524, 2018).

11 PSMFC, Pacific States Marine Fisheries Commission,. PIT tag information system. Interactive database maintained by the Pacific States Marine Fisheries Commission, Portland, Oregon, www.ptagis.org, accessed October, 2019. (2019).

12 Crozier, L. G. *et al.* Snake River sockeye and Chinook salmon in a changing climate: implications for upstream migration survival during recent extreme and future climates. *PLoS ONE* **15**, e0238886. https://doi.org/0238810.0231371/journal.pone.0238886 (2020).

13 USGS, U.S. Geological Society, Water Resources,. Surface-water data for Idaho. (http://waterdata.usgs.gov/id/nwis/. Accessed May 2019, 2019).

14 PRISM Climate Group. Oregon State University, http://prism.oregonstate.edu, accessed May, 2019. (2019).

15 U.S. Army Corps of Engineers (ACOE), Northwestern Division Bureau of Reclamation

& Administration, P. N. R. B. P. Columbia River System Operations Draft Environmental Impact Statement, February 2020. *DOE/EIS-0529* (2020).

16 DART. Columbia River Data Access in Real Time. *http://www.cbr.washington.edu/dart/dart.html, accessed June 2019* (2019).

17 NCEI, National Centers for Environmental Information. *Extended Reconstructed Sea Surface Temperature (ERSST) v5*, <https://www.pfeg.noaa.gov/products/PFELData/upwell/monthly/upindex.mon> (2019).

18 PFEL, Pacific Fisheries Environmental Laboratory. *PFEL Global 1-degree Upwelling Index*, <https://www.pfeg.noaa.gov/products/PFELData/upwell/monthly/upindex.mon> (2019).

19 Zabel, R. W., Scheuerell, M. D., McClure, M. M. & Williams, J. G. The interplay between climate variability and density dependence in the population viability of Chinook salmon. *Conservation Biology* **20**, 190-200 (2006).

20 Gelman, A. *et al. Bayesian Data Analysis*. 661 (CRC Press, 2014).

21 Su, Y.-S. & Yajima, M. R2jags: Using R to Run 'JAGS'. R package version 0.5-7. https://CRAN.R-project.org/package=R2jags. (2015).

22 R Core Team. R version 3.6.2: A language and environmental for statistical computing. R Foundation for Statistical Computing, Vienna, Austria. USBN 3-900051-0700, available at www.R-project.org. Version 3.0.1. (2019).

23 Vehtari, A., Gelman, A. & Gabry, J. Practical Bayesian model evaluation using leave-one-out cross-validation and WAIC. *Statistics and Computing* **27**, 1413-1432, doi:doi: 10.1007/s11222-016-9696-4 (2017).

24 Zabel, R. W. *et al.* Comprehensive passage (COMPASS) model: a model of downstream migration and survival of juvenile salmonids through a hydropower system. *Hydrobiologia* **609**, 289-300, doi:10.1007/s10750-008-9407-z (2008).

25 Sandford, B. P. & Smith, S. G. Estimation of smolt-to-adult return percentages for Snake River Basin anadromous salmonids, 1990-1997. *Journal of Agricultural Biological and Environmental Statistics* **7**, 243-263, doi:10.1198/10857110260141274 (2002).

26 Burnham, K. P. & Anderson, D. R. *Model selection and inference--a practical information-theoretic approach*. 2nd edn, (Springer-Verlag, 2002).

27 Wood, S. N. *Generalized Additive Models: An introduction with R, second edition.* (Chapman and Hall/CRC, 2017).

28 Crozier, L., Dorfmeier, E., Marsh, T., Sandford, B. & Widener, D. Refining our understanding of early and late migration of adult Upper Columbia spring and Snake River spring/summer Chinook salmon: passage timing, travel time, fallback and survival. (Report of research by Fish Ecology Division, Northwest Fisheries Science Center. Available at https://www.nwfsc.noaa.gov/contact/display_staffprofilepubs.cfm?staffid=1471, 2016).

29 Crozier, L. G., Bowerman, T. E., Burke, B. J., Keefer, M. L. & Caudill, C. C. High-stakes steeplechase: a behavior-based model to predict individual travel times through diverse migration segments. *Ecosphere* **8**, e01965-n/a, doi:10.1002/ecs2.1965 (2017).

30 Brekke, L., Kuepper, B. & Vaddey, S. Climate and Hydrology Datasets for Use in the RMJOC Agencies' Longer-Term Planning Studies: Part 1 - Future Climate and Hydrology Datasets, available at https://www.usbr.gov/pn/climate/planning/reports/index.html. (2010).

31 Hamlet, A. F. *et al.* An Overview of the Columbia Basin Climate Change Scenarios Project: Approach, Methods, and Summary of Key Results. *Atmosphere-Ocean* **51**, 392-415, doi:10.1080/07055900.2013.819555 (2013).
