# Supplementary material for: Climate change threatens Chinook salmon throughout their life cycle
Source: Commun Biol. 2021 Feb 18;4:222. doi: 10.1038/s42003-021-01734-w (PMC7892847; doi:10.1038/s42003-021-01734-w)
Supplement: Supplementary file 3 — Description of Additional Supplementary Files [file 42003_2021_1734_MOESM3_ESM.pdf]

Description of Additional Supplementary Files

File Name: Supplementary Data 1
Description: The Supplementary Data file contains the data used to make figures 2, 4, 5, 6, 7 and 9. Data is presented in columns for each variable with the year in rows for figures 2 (climate drivers), 4 (population abundance), 5 (the number of simulations in which the population passed the quasi-extinction threshold within a scenario), and 9 (annual population abundance estimates with both empirical and life cycle model results). Data is presented as a single result for each simulation in rows for figures 6 (year the population passed the quasi-extinction threshold) and 7 (ratio of spanner abundance in the two time periods).
