# Supplementary material for: Climate change threatens Chinook salmon throughout their life cycle
Source: Commun Biol. 2021 Feb 18;4:222. doi: 10.1038/s42003-021-01734-w (PMC7892847; doi:10.1038/s42003-021-01734-w)
Supplement: Supplementary file 5 — Reporting summary [file 42003_2021_1734_MOESM5_ESM.pdf]

nature research

Corresponding author(s): Lisa Crozier

Last updated by author(s): Nov 17, 2020

Reporting Summary

Nature Research wishes to improve the reproducibility of the work that we publish. This form provides structure for consistency and transparency in reporting. For further information on Nature Research policies, see our Editorial Policies and the Editorial Policy Checklist.

Statistics

For all statistical analyses, confirm that the following items are present in the figure legend, table legend, main text, or Methods section.

n/a	Confirmed	
☒	☐	The exact sample size (n) for each experimental group/condition, given as a discrete number and unit of measurement
☒	☐	A statement on whether measurements were taken from distinct samples or whether the same sample was measured repeatedly
☐	☒	The statistical test(s) used AND whether they are one- or two-sided<br>*Only common tests should be described solely by name; describe more complex techniques in the Methods section.*
☐	☒	A description of all covariates tested
☐	☒	A description of any assumptions or corrections, such as tests of normality and adjustment for multiple comparisons
☐	☒	A full description of the statistical parameters including central tendency (e.g. means) or other basic estimates (e.g. regression coefficient) AND variation (e.g. standard deviation) or associated estimates of uncertainty (e.g. confidence intervals)
☒	☐	For null hypothesis testing, the test statistic (e.g. F, t, r) with confidence intervals, effect sizes, degrees of freedom and P value noted<br>*Give P values as exact values whenever suitable.*
☐	☒	For Bayesian analysis, information on the choice of priors and Markov chain Monte Carlo settings
☐	☒	For hierarchical and complex designs, identification of the appropriate level for tests and full reporting of outcomes
☒	☐	Estimates of effect sizes (e.g. Cohen's d, Pearson's r), indicating how they were calculated

Our web collection on statistics for biologists contains articles on many of the points above.

Software and code

Policy information about availability of computer code

Data collection	NA
Data analysis	R x64 3.6.2<br>Rstudio 1.2.5001<br>R code for running the model is available on GitHub (lisa439/LCM)

For manuscripts utilizing custom algorithms or software that are central to the research but not yet described in published literature, software must be made available to editors and reviewers. We strongly encourage code deposition in a community repository (e.g. GitHub). See the Nature Research guidelines for submitting code & software for further information.

Data

Policy information about availability of data

All manuscripts must include a data availability statement. This statement should provide the following information, where applicable:

- Accession codes, unique identifiers, or web links for publicly available datasets
- A list of figures that have associated raw data
- A description of any restrictions on data availability

All of the raw survival data used in this analysis are publicly available for download from PIT Tag Information System (www.PTAGIS.org).
All survival estimates made from the raw data are published in other sources, listed in Table S1.
Redd counts are available upon request from Idaho Department of Fish and Game (IDFG.idaho.gov) for all but one population, and from the Nez Perce Tribe for the Secesh River population (https://www.cbfish.org/Document.mvc/Viewer/P165414). Population estimates from redd counts were published in Ford et al. (2016) (https://www.nwfsc.noaa.gov/publications/scipubs/display_doctrack_allinfo.cfm?doctrackmetadataid=8623) and are available at https://www.webapps.nwfsc.noaa.gov/apex/f?p=261:HOME::::::#.

Environmental data are all publicly available from sources cited in Supplementary Tables 1 and 3.

Field-specific reporting

Please select the one below that is the best fit for your research. If you are not sure, read the appropriate sections before making your selection.

☐ Life sciences ☐ Behavioural & social sciences ☒ Ecological, evolutionary & environmental sciences

For a reference copy of the document with all sections, see nature.com/documents/nr-reporting-summary-flat.pdf

Ecological, evolutionary & environmental sciences study design

All studies must disclose on these points even when the disclosure is negative.

Study description	Study is based on spawner abundance estimates from redd counts and stage-specific survival estimates for Chinook salmon from the Snake River spring/summer ESU.
Research sample	These populations were chosen because they are not influenced by hatchery production and they have extensive individual tagging of wild fish prior to the smolt migration (described in Lamb et al 2018).
Sampling strategy	Protocol and Methods redd surveys are available at https://fortress.wa.gov/dfw/score/score/; http://odfwrecoverytracker.org/metadata/; https://www.monitoringmethods.org/Protocol/Details/159; https://www.monitoringmethods.org/Protocol/Details/235.
Data collection	NA
Timing and spatial scale	The analysis used primarily data from 2000-2015, when there were sufficient tagging rates and detection ability at Bonneville Dam for reliable estimates of smolt to adult return. Longer time frames are possible for redd counts and parr to smolt survival. However, management of the Columbia River hydrosystem, especially how many fish were transported downstream and levels of spill, were very different in the 1990s, so we considered this period not to be representative of the near future
Data exclusions	We did not exclude any available estimates of survival
Reproducibility	The model results are easily reproducible
Randomization	NA
Blinding	NA

Did the study involve field work? ☐ Yes ☒ No

Reporting for specific materials, systems and methods

We require information from authors about some types of materials, experimental systems and methods used in many studies. Here, indicate whether each material, system or method listed is relevant to your study. If you are not sure if a list item applies to your research, read the appropriate section before selecting a response.

Materials & experimental systems

n/a	Involved in the study
☒	☐ Antibodies
☒	☐ Eukaryotic cell lines
☒	☐ Palaeontology and archaeology
☒	☐ Animals and other organisms
☒	☐ Human research participants
☒	☐ Clinical data
☒	☐ Dual use research of concern

Methods

n/a	Involved in the study
☒	☐ ChIP-seq
☒	☐ Flow cytometry
☒	☐ MRI-based neuroimaging
